# Relationship between bullying victimisation and post-traumatic stress disorder among public junior secondary school students in Abeokuta, Nigeria

**DOI:** 10.1192/bjo.2021.627

**Published:** 2021-06-18

**Authors:** Sewanu Awhangansi, Titilayo Salisu, Oluwayemisi Akanji, Adeniran Okewole, Oladipo Sowunmi, Sunday Amosu, Increase Adeosun, Olugbenga Owoeye

**Affiliations:** 1 Leicestershire partnership NHS Trust; 2 Federal University of Agriculture (FUNAAB); 3 Bowen University Teaching Hospital; 4 Neuropsychiatric Hospital Aro; 5 Babcock University Medical School; 6 Federal Neuropsychiatric Hospital

## Abstract

**Aims:**

To determine the relationship between bullying victimization and PTSD among students attending public Junior Secondary Schools in Abeokuta. The Prevalence of Bullying victimization and PTSD as well as some socio-demographic correlates were also assessed.

**Method:**

About 411 junior students from five randomly selected public secondary schools were approached for the study and given consent forms to take home to their parents/guardians. Those who subsequently returned signed consent forms and who gave assent to participate in the study were administered the Socio-demographic questionnaire and the Multidimensional Peer Victimisation Scale (MDPVS). They were thereafter interviewed with the PTSD module of the MINI KID.

**Result:**

A total of 351 students completed the study to yield a response rate of 85.4%. The age range of the respondents was 9–17 years with mean (SD) of 12.48 (1.50) years. The gender distribution was 49.3% males and 50.7% females. 68.7% of the respondents were from a monogamous home, 22.2% had divorced parents, 74.3% lived with both parents, and 6% reported being an only child. 14.8% of the respondents reported having experienced higher levels (moderate & high) of victimization by peers. The mean score of the overall bullying victimization level was 9.6 (±6.5). Verbal victimization subscale had the highest mean score of 3.2 (±2.0), while physical victimization had the lowest mean of 1.9 (±2.1). Seventy (19.9%) students admitted to the experience of a significant traumatic event, with only 7.1% of these meeting the current diagnosis of PTSD in the past month. There was no statistically significant association between bullying victimization and PTSD (χ2 = 2.666; df = 2; p = 0.261). Traumatic event experience was however significantly associated with high levels of bullying victimization experience (χ2 = 4.266; p = 0.039). None of the assessed socio-demographic, familial or self-perceptual factors was found to be significantly associated with either bullying victimization or PTSD.

**Conclusion:**

The experience of bullying victimization among secondary school students remains a prevailing problem in our local setting, as it is across the globe. Verbal bullying is the most common while physical bullying is the least common peer victimization experience in this study. The study points out that PTSD among high school students in our environment may be more prevalent than had previously been reported. Given the high rates of peer victimization experiences reported by students, there is a need for policy changes to make the school environment safer for students, thereby promoting their mental health.

